# Congenital disorders of glycosylation: narration of a story through its patents

**DOI:** 10.1186/s13023-023-02852-w

**Published:** 2023-08-29

**Authors:** Maria Monticelli, Tania D’Onofrio, Jaak Jaeken, Eva Morava, Giuseppina Andreotti, Maria Vittoria Cubellis

**Affiliations:** 1 Department of Biology, University of Napoli “Federico II”, Complesso Universitario Monte Sant’Angelo, Via Cinthia, Napoli, 80126 Italy; 2 Institute of Biomolecular Chemistry ICB, CNR, Via Campi Flegrei 34, Pozzuoli, 80078 Italy; 3 Center of Metabolic Diseases, KU Leuven, Leuven, Belgium; 4 Department of Clinical Genomics and Laboratory of Medical Pathology, Mayo Clinic, Rochester, MN USA; 5 Stazione Zoologica “Anton Dohrn”, Villa Comunale, Naples, Italy

**Keywords:** Congenital disorder(s) of glycosylation, CDG, Rare disease, Intellectual property, Patent, Drug Discovery, Diagnosis

## Abstract

Congenital disorders of glycosylation are a group of more than 160 rare genetic defects in protein and lipid glycosylation. Since the first clinical report in 1980 of PMM2-CDG, the most common CDG worldwide, research made great strides, but nearly all of them are still missing a cure. CDG diagnosis has been at a rapid pace since the introduction of whole-exome/whole-genome sequencing as a diagnostic tool. Here, we retrace the history of CDG by analyzing all the patents associated with the topic. To this end, we explored the Espacenet database, extracted a list of patents, and then divided them into three major groups: (1) Drugs/therapeutic approaches for CDG, (2) Drug delivery tools for CDG, (3) Diagnostic tools for CDG. Despite the enormous scientific progress experienced in the last 30 years, diagnostic tools, drugs, and biomarkers are still urgently needed.

**Supplementary Information:**

The online version contains supplementary material available at 10.1186/s13023-023-02852-w.

## Background

According to the Orphan Drug Act, a rare disease is a disease or condition that impacts fewer than 200,000 people in the US [1, 2]. European Union considers as rare a disease affecting fewer than 5 people in 10,000 [3]. Over 6,000 rare diseases have been identified, affecting 8–10% of the world’s population [4–6].

The low prevalence of each disease, the wide diversity of symptoms and signs that vary not only from disease to disease but also from patient to patient suffering from the same condition, the limited knowledge and unclear underlying biology of many rare diseases, the lack of sufficient medical expertise as well as the lack of rare disease awareness and adequate financial resources, still pose significant challenges to patients, clinicians, and scientists [7–9].

Congenital disorders of glycosylation (CDG) are a varied group of rare genetic diseases characterized by protein and lipid hypoglycosylation [10, 11]. In 1980, prof. Jaak Jaeken described a new neurological disorder in twin girls [12]. This disorder’s clinical features, stages, progression, and biochemical analyses were depicted in 1991 [13]. The genetic evidence that phosphomannomutase 2 deficiency was the basis for the disease defined as “carbohydrate-deficient glycoprotein syndrome” was obtained in 1997 [14]. The scientific CDG community has come a long way since then, with an ever-growing number of new patients, new CDG, clinicians and researchers committed to this field, and a large body of research papers related to clinical, genetic, biological and biochemical results, diagnosis, and treatments. However, most CDG still do not have a cure, and a correct diagnosis is often challenging to obtain in a reasonable time [7].

This mini-review looks at CDG through Intellectual Property (IP) indicators. Many reviews on CDG have been published (about 40 only from 2021 to today - Pubmed access on 24th April 2023). Most focus on clinical signs and management, others on pathophysiology or treatment options. Here, we narrate the story of CDG through the associated patents.

## Results

### Research outcomes

For this review, we used a combination of keywords related to CDG to search the Espacenet database [15]. Queries are specified in Table 1, and the original files are available as Supplementary Files 1, 2, 3 and 4.

**Table 1:** Search strategies for identifying patents for CDG on EspaceNet (https://worldwide.espacenet.com/)

#	Query	Number of patents	Date of the research	Supplementary file name
1	(nftxt = “congenital” AND nftxt = “disorder*” AND nftxt = “glycosylation” AND nftxt = “cdg”) NOT nftxt = “CFTR”	157	18/02/2023	Supplementary File 1- Espacenet_search_result_20230218_1749
2	Phosphomannose isomerase deficiency	238	08/05/2023	Supplementary File 2 - Espacenet_search_result_20230508_2340
3	(nftxt = “PMM2-CDG” OR nftxt = “MPI-CDG” OR nftxt = “ALG6-CDG” OR nftxt = “ALG3-CDG” OR nftxt = “DPM1-CDG” OR nftxt = “MPDU1-CDG” OR nftxt = “ALG12-CDG” OR nftxt = “ALG8-CDG” OR nftxt = “ALG2-CDG” OR nftxt = “MGAT2-CDG” OR nftxt = “MOGS-CDG” OR nftxt = “Leukocyte adhesion deficiency type II” OR nftxt = “COG7-CDG” OR nftxt = “SLC35A1-CDG” OR nftxt = “COG1-CDG” OR nftxt = “COG8-CDG” OR nftxt = “COG5-CDG” OR nftxt = “COG4-CDG” OR nftxt = “TMEM165-CDG” OR nftxt = “COG6-CDG” OR nftxt = “SLC35A2-CDG” OR nftxt = “SLC39A8-CDG” OR nftxt = “CCDC115-CDG” OR nftxt = “TMEM199-CDG” OR nftxt = “DPAGT1-CDG” OR nftxt = “ALG1-CDG” OR nftxt = “ALG9-CDG” OR nftxt = “DK1-CDG” OR nftxt = “RFT1-CDG” OR nftxt = “DPM3-CDG” OR nftxt = “ALG11-CDG” OR nftxt = “SRD5A3-CDG” OR nftxt = “DDOST-CDG” OR nftxt = “ALG13-CDG” OR nftxt = “PGM1-CDG” OR nftxt = “Congenital muscular dystrophy with intellectual disability and severe epilepsy” OR nftxt = “STT3A-CDG” OR nftxt = “STT3B-CDG” OR nftxt = “SSR4-CDG” OR nftxt = “CAD-CDG” OR nftxt = “MAN1B1-CDG” OR nftxt = “B4GALT1-CDG” OR nftxt = “XYLT1-CDG” OR nftxt = “PGM3-CDG” OR (nftxt = “glycosylation defect*” OR ctxt = “carbohydrate associated protein*” OR (ctxt = “carbohydrate deficient” AND ctxt = “glycoprotein” AND ctxt = “syndrome*”) OR (ctxt = “congenital” AND ctxt = “disorder*” AND ctxt = “glycosylation”))) NOT nftxt = “cftr”	392	21/05/2023	Supplementary File 3 - Espacenet_search_result_20230521_1219
4	((ctxt = “PMM2-CDG” OR nftxt = “phosphomannomutase deficiency”) OR (ctxt = “MPI-CDG” OR nftxt = “phosphomannose isomerase deficiency” OR (ctxt = “PGM*-CDG” OR nftxt = “phosphoglucomutase deficiency”) OR ctxt = “leukocyte adhesion deficiency”) OR (ctxt = “glycosylation defect*” OR ctxt = “carbohydrate associated protein*” OR (ctxt = “carbohydrate deficient” AND ctxt = “glycoprotein” AND ctxt = “syndrome*”) OR (ctxt = “congenital” AND ctxt = “disorder*” AND ctxt = “glycosylation”))) NOT nftxt = “ctfr”	415	21/05/2023	Supplementary File 4 - Espacenet_search_result_20230521_1222

We manually analyzed the extracted lists. First, we merged the lists based on the title. Then, patents were selected based on the title and the bibliographic data. We only considered English-written patents that included the original documents. In the cases where the original document was not in English, but the patent had another publication number whose original document was in English, we used the last one. We also considered patents written in other languages whose original document contained an abstract written in English. Refinement of the results included eliminating duplicates and defining a final subset containing 43 patents (Supplementary File 5).

A final reading of the complete original documents allowed a classification in the following classes: (1) Drugs/therapeutic approaches for CDG, (2) Drug delivery tools for CDG, (3) Diagnostic tools for CDG, (4) Production/modification/characterization of glycoconjugates. We discharged the last group, which is not strictly specific to CDG.

**Fig. 1:**
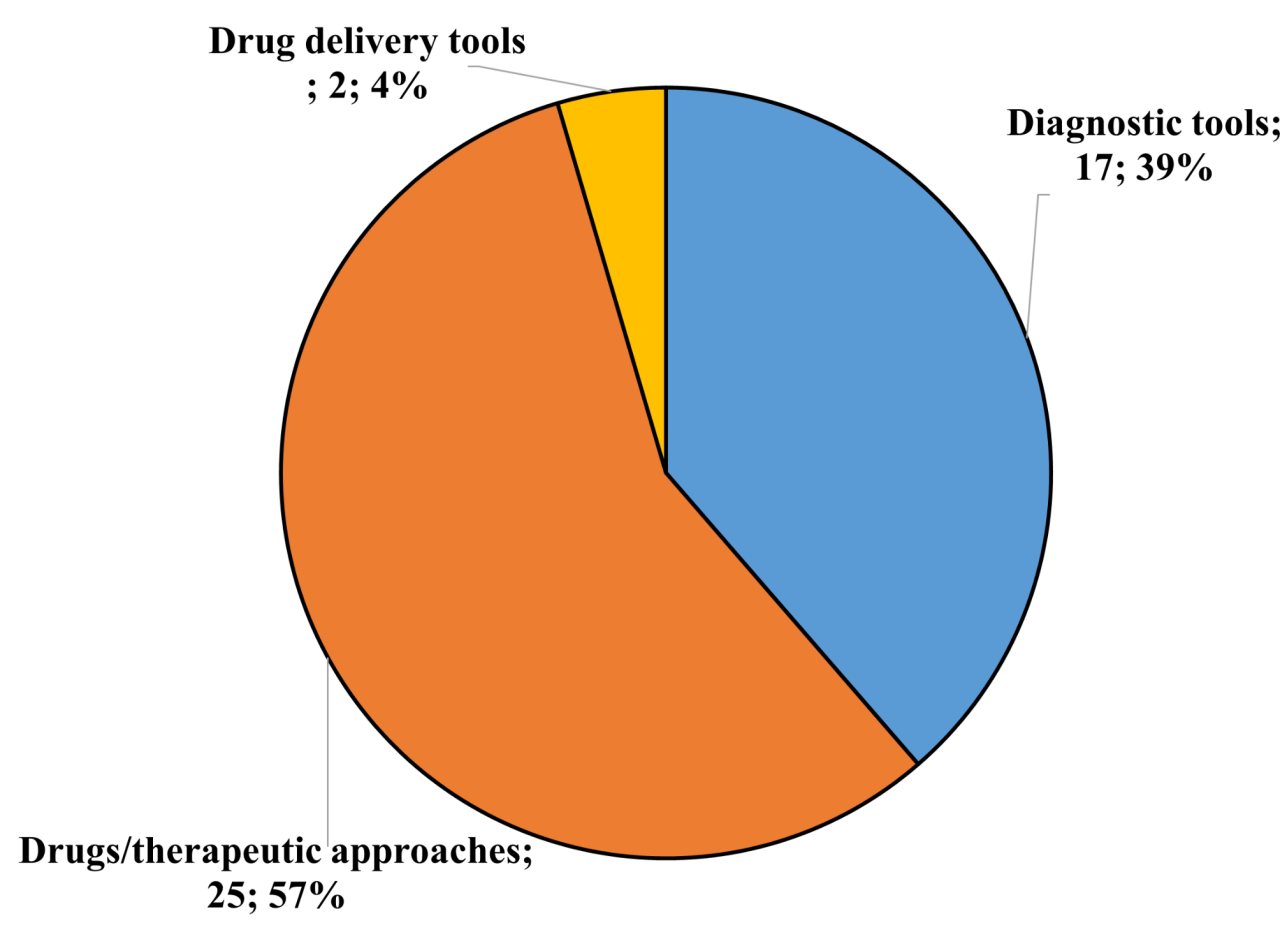
Classification of the patents derived from the research. For each class, the total number and the percentage are shown

Figure 1 shows the distribution of the final list of 43 patents − 25 in class 1 (Drugs/Therapeutic Approaches), 2 in class 2 (Drug Delivery Tools), and 17 in class 3 (Diagnostic Tools) - that were further analyzed and commented on. One patent (EP2905621A1) overlaps both classes 1 and 3.

**Fig. 2:**
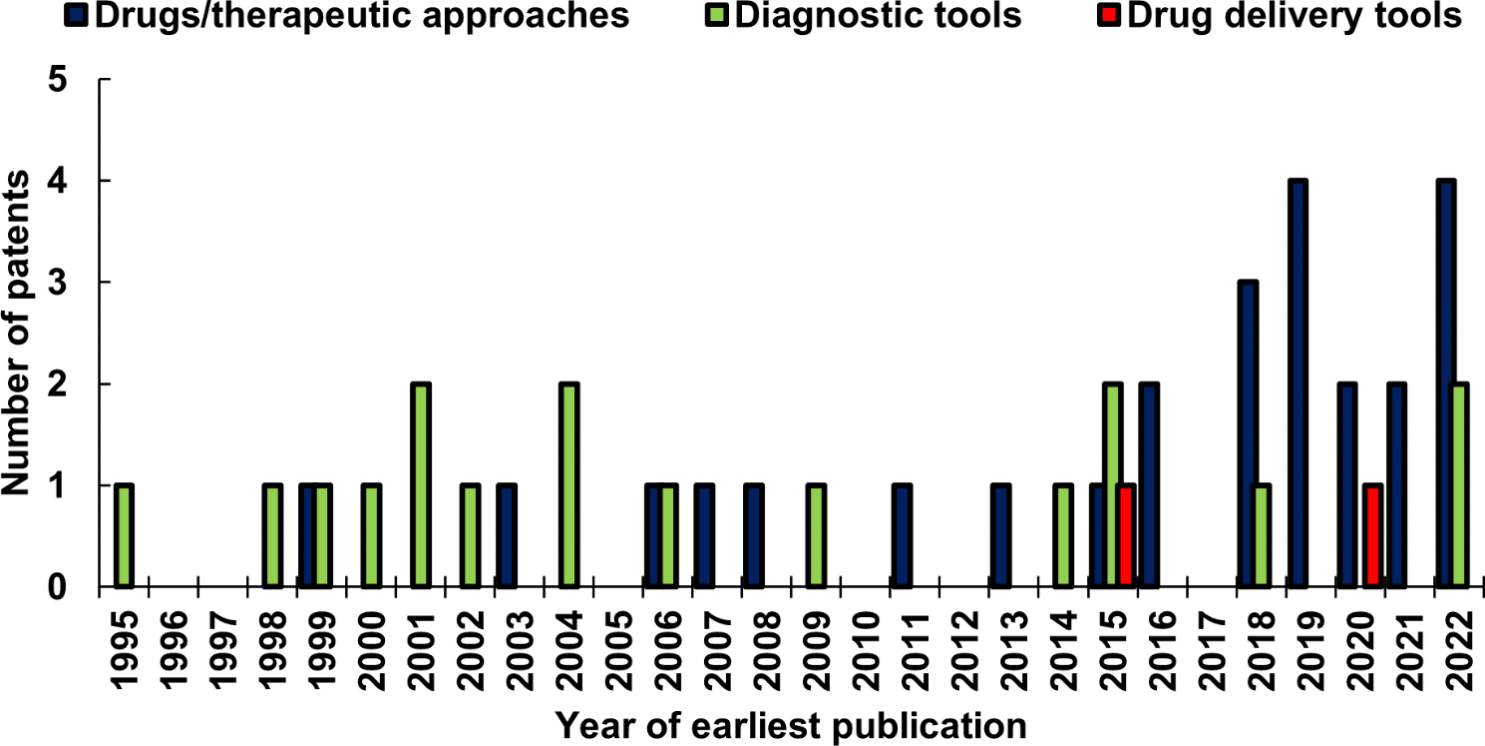
Temporal distribution of patents’ publications

The first clinical description of CDG dates from 1980, and the first patent regarding CDG from 1995. Nevertheless, according to Google Scholar, in the period 1980–1995, 27 papers were published citing the findings by Jaak Jaeken; among these, only three belonged to the 1980–1990 decade [16–18]. In 1991, Jaak Jaeken depicted the newly identified syndrome’s clinical and biochemical phenotype, naming it “carbohydrate-deficient glycoprotein syndrome” [13]. Until 1995, this breakthrough was cited by 33 papers, thus representing a pivotal point after an almost silent decade. In this period, the number of identified patients and their symptoms started to multiply [19–23], and the first description of a different carbohydrate-deficient glycoprotein syndrome appeared, defined as “type II” [24]. Since then, the CDG family has rapidly expanded and currently counts more than 160 CDG [25]. All the patents were granted in the last three decades (Fig. 2).

On the one hand, those concerning the diagnostic tools are homogeneous in the considered period. However, the variable phenotypic spectrum and the absence of a genotype-phenotype correlation still hampers a fast and correct diagnosis. Moreover, the role of genomic variants in the development of the disease is a critical issue that has not yet been extensively investigated [26–29].

On the other hand, as expected, the patents regarding drugs and drug delivery tools were produced mainly in the last decade due to the increasing knowledge of the basic molecular mechanisms of the diseases, the development of cell models, the identification of biomarkers, and the development or improvement of biotechnologies such as genetic manipulation.

**Fig. 3:**
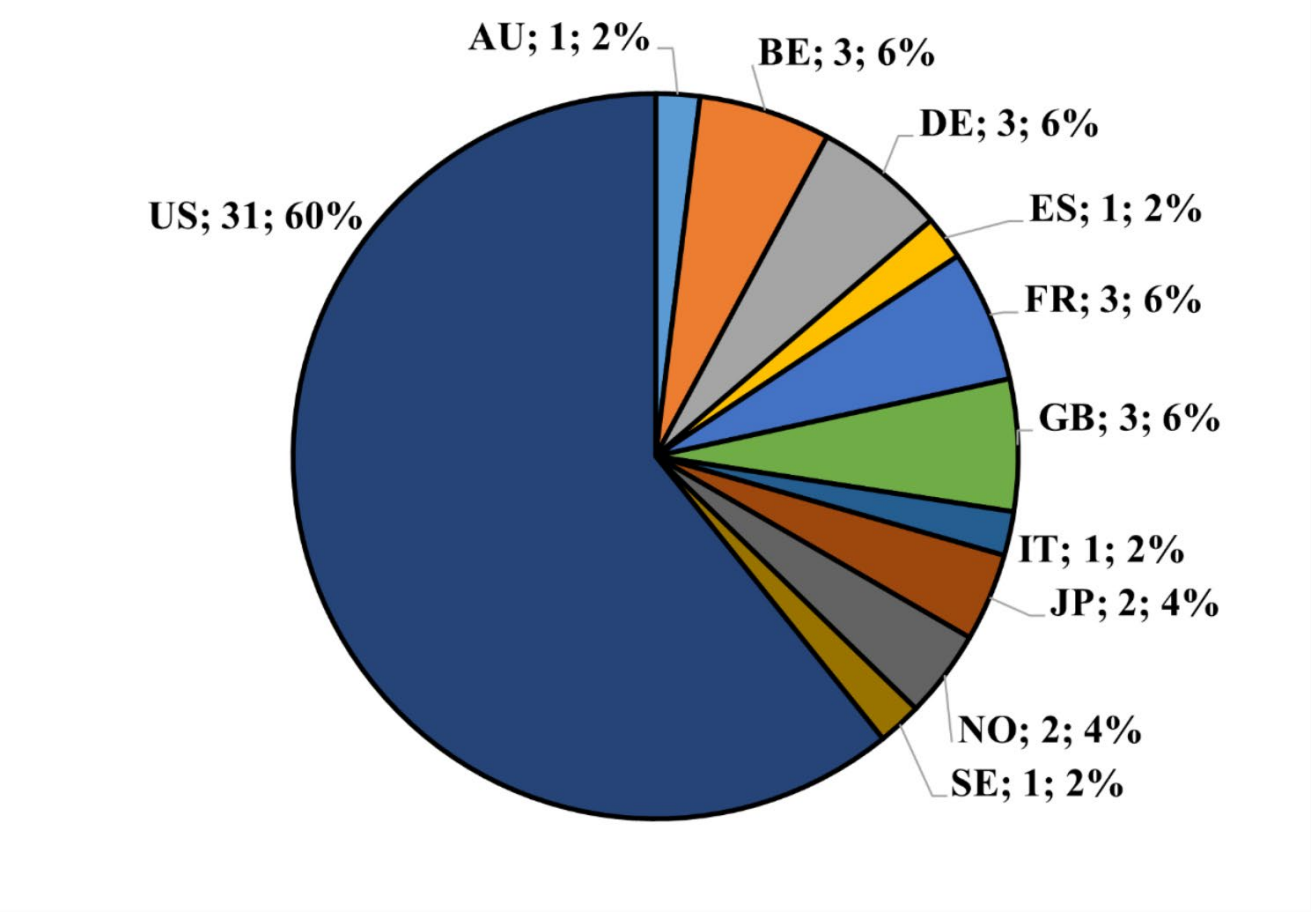
Geographical distribution of patents’ applicants. US: United States of America; AU, Australia; BE: Belgium; DE: Germany; ES: Spain; FR: France, GB: Great Britain; IT, Italy; JP: Japan; NO: Norway; SE, Sweden. For each country, the total number and the percentage are shown

Figure 3 shows the distribution of patents according to the applicant’s country. Most applicants were from the US (31; 60%). According to the data provided by the World Intellectual Property Organization (WIPO), “More than 85% of all patent filings in 2021 occurred in the IP offices of China, the US, Japan, the Republic of Korea and the EPO (European Patent Office). China accounted for 46.6% of the world total.“ However, according to this site, applicants from China filed firstly in ‘Computer technology’, secondarily in ‘Digital communication’, and thirdly in ‘Electrical machinery, apparatus, energy’; US applicants filed mostly in ‘Computer technology’ too, but their second top technology for applications is ‘Medical technology’ [30]. Even if our results represent a total production over three decades, this aligns with WIPO data.

### Drugs/therapeutic approaches and drug delivery tools for CDG

Table 2 lists patents on drugs and therapeutic approaches, while Table 3 lists those related to drug delivery tools.

**Table 2:** Drugs/therapeutic approaches for CDG.

Patent id/Title/ earliest publication	Compound(s)	Tested	Defect/CDG/protein	Category/rationale
DE19758059A1 WO9933474A1 The use of mannose for combating protein loss enteropathy (1999)	Mannose or mannose-containing sugar which releases mannose in the gastro-intestinal tract	Mannose supplementation was successfully tested on CDGIb patient’ fibroblasts (2 [^3^ H]mannose incorporation into glycoconjugates was measured and normal glycosylation of glycoproteins was recorded). Mannose was given orally to the patient too. The clinical symptoms disappeared and transferrin glycoprofile normalized. [35, 36, 37]	Protein N-glycosylation: • MPI-CDG Phosphomannose isomerase	Sugar supplementation therapy Mannose provided by the diet becomes the only source of mannose 6-phosphate
EP1521761A2 WO03104247A2 Treatment of congenital disorders of glycosylation (CDG) using mannose (2003)	Hydrophobically masked derivatives of mannose-1P	Enzymatic tests have been conducted to demonstrate that protecting groups were cleaved to restore the original mannose-1P. [48]	Protein N-glycosylation: • PMM2-CDG Phosphomannomutase 2	Substrate replacement therapy The hydrophobically masked mannose 1-phosphate derivatives having an increased lipophilicity can cross the cell membrane. Once inside the cell, endogenous nonspecific enzymes should ensure the release of the free monophosphate sugar
FR2897779A1 FR2897779B1 US2009054353A1 Drug composition, useful as cellular sources of mannose-1-phosphate against carbohydrate deficient glycoprotein type I syndrome, comprises excipient and active ingredient e.g. mono(alpha-D-mannopyranosyl-1) phosphate (2007)	Mono-(mannopyranosyl-1), di(mannopyranosyl-1) and tri(mannopyranosyl-1) phosphates	Lymphoblast cells derived from a control subject and a CDG-Ia patient were used. Inhibition of 2 [^3^ H]mannose incorporation into glycoconjugates was recorded. The toxicity of prodrugs has also been assessed. [49]	Protein N-glycosylation: • PMM2-CDG Phosphomannomutase 2	Substrate replacement therapy
WO2011116355A2 WO2011116355A3 Benzoisothiazolones as inhibitors of phosphomannose isomerase (2011)	Benzoisothiazolones	The potency and selectivity of the compounds were assessed running enzymatic assays on human PMI and PMM2. Fresh plasma or liver microsomes (mouse) were used for stability assays. Cellular assay was conducted on HeLa cells (2 [^3^ H]mannose incorporation into glycoconjugates was measured). C57BL/6 mice were used for in vivo pharmacokinetics. [50]	Protein N-glycosylation: • PMM2-CDG Phosphomannomutase 2	Phosphomannose isomerase (PMI) inhibitors The inhibition of PMI can push the flux of mannose-6P towards the production of mannose-1P
EP3275863A1 Compounds for treating congenital disorders of glycosylation (2018)	Amide and urea derivatives (e.g.: 3-(3-Chloro-phenyl)-1,1-di-pyridin-2-yl-urea and 1,3-Bis-(3-chlorophenyl)-urea)	Pure, homodimeric WT-PMM2 and mutants (p.Val44AIa, p.Asp65Tyr, p.Arg123Gln, p.Arg141 His, p.Arg162Trp, p.Thr237Met, p.Cys241Ser) were analysed by Differential Scanning Fluorimetry. Patient-derived fibroblasts (p.Arg141His/p.Asp65Tyr), (p.Pro113Leu/p.Pro113Leu), (p.Arg141His/p.Arg162Trp) and (c.640-9T > G/p.Thr237Met) were transduced with their own folding or oligomerization variant, that is p.Asp65Tyr, p.Pro113Leu, p.Arg162Trp, and p.Thr237Met respectively. These cellular models were used to evaluate PMM2 activity after the treatment with selected drugs. [56]	Protein N-glycosylation: • PMM2-CDG Phosphomannomutase 2	Pharmacological chaperone therapy The drug binds to and stabilize wt and mutated PMM2s. When the variant does not affect the enzymatic activity but only causes a loss of stability, a PC prevents the premature degradation of the mutated protein
WO2020040831A1 Methods for treating congenital disorders of glycosylation (2020)	α-cyano-4-hydroxycinnamic acid, epalrestat, rhetsinine, theasinensin, suramin	Drug screen/test was conducted in: -yeast models of PMM2-CDG (pACT1-F126L – p.Phe126Leu, pSEC53-V238M – p.Val238Met, and pSEC53-F126L -p.Phe126Leu haploids and pACT1-F126L/pACT1-R148H – p.Phe126Leu/p.Arg148His, pSEC53-V238M/pSEC53-R148H – p.Val238Met/p.Arg148His, and pSEC53-F126L/pSEC53-R148H – p.Phe126Leu/p.Arg148His heterozygous diploids). - a p.Phe119Leu variant strain, orthologous p.Phe125Leu in worms - WT and PMM2 (GM20942-p.Arg141His/p.Phe119Leu) compound heterozygous fibroblasts. [58, 59]	Protein N-glycosylation: • PMM2-CDG Phosphomannomutase 2	Aldose reductase inhibitors Aldose reductase inhibitors may reduce the flux of glucose through the polyol pathway, which can lead to inhibition of tissue accumulation of sorbitol and fructose and prevention of reduction of redox potentials
US2022017535A1 Inhibitors of aldose reductase (2020)	A novel family of aldose reductase inhibitors	In vitro studies measuring aldose reductase enzymatic inhibition were conducted. Ex vivo studies on an isovolumic isolated rat heart preparation (male Wistar rats) were also conducted.	Protein N-glycosylation • PMM2-CDG Phosphomannomutase 2	Aldose reductase inhibitors
CA3153108A1 WO2021071965A1 Aldose reductase inhibitors for treatment of phosphomannomutase 2 deficiency (2021)	Zopolrestat, ponalistat, epalrestat, sobinil or sorbinol, mirlistat, AND-138, CT-112 (Risarestat), zopostat, denastat, BAL-AR18, AD-5467, M-79,175, torilista, alconil, statil, berberine or SPR-210	PMM2 enzymatic assay was conducted on four protein extracts from PMM2-CDG patient-derived fibroblasts (p.Phe138Ser/p.Arg141His, p.Arg141His/p.Pro113Leu, p.Arg141His/p.Phe119Leu p.Arg141His/p.Asn216Ile) treated with the drugs.	Protein N-glycosylation: • PMM2-CDG Phosphomannomutase 2	Aldose reductase inhibitors Single or combined therapy (with a second aldose reductase inhibitor, an antioxidant, or both)
GB2597315A Use of cannabidiol in the treatment of seizures associated with rare epilepsy syndromes related to genetic abnormalities (2022)	Cannabidiol	CBD was able to significantly reduce the number of seizures in one ALG11-CDG patient.	Protein N-glycosylation: • ALG11-CDG ALG11 Alpha-1,2-Mannosyltransferase	Treatment of seizures associated with rare epilepsy syndromes Single or combined therapy with one or more other anti-epileptic drugs. The combined administration can be done sequentially or simultaneously
EP2905621A1 Means and methods for diagnosing and treating cdg caused by a deficiency of PGM1 (2015)	Galactose and uridine	Galactose was added to culture medium of fibroblasts from patients. An enhancement of the glycosylation was recorded on ICAM-1 and Glyc-ER-GFP. The effect on Glyc-ER-GFP was further enhanced by the addition of uridine to the culture medium. Six patients received dietary supplementation with lactose or galactose. Transferrin IEF profiles showed a substantial improvement of glycosylation after dietary intake of galactose. Other clinical signs have been monitored too. [79]	Disorder of multiple glycosylation pathways: • PGM1-CDG Phosphoglucomutase 1	Sugar supplementation therapy
WO2016028894A1 Treatment of glycosylation deficiency diseases (2016)	Uridine prodrug (e.g.: uridine triacetate) and sugars	A patient diagnosed with a CDG by detection of a variation affecting glycosylation by reducing availability of a UDP-sugar was treated with uridine triacetate. One GNE-myopathy patient, one PGM1-CDG patient, one DGAPT1-CDG patient were treated with uridine triacetate in combination with N-acetylmannosamine, D-galactose, N-acetylglucosamine respectively. The combined therapies produced a clinical improvement better than the sugars alone.	Disorder of multiple glycosylation pathways: • PGM1-CDG Phosphoglucomutase 1 • GNE-CDG UDP-GlcNAc 2-epimerase/ManNAc kinase Protein N-glycosylation: • DPAGT1-CDG UDP-GlcNAc: dolichylphosphate N-Acetylglucosaminephosphotransferase 1	Combined therapy (uridine prodrug and sugars). The administration of uridine prodrug increases the intracellular UTP. The coadministration of the specific sugar or its precursor, causes increased intracellular concentrations of the UDP-sugar
EP3806866A1 EP3806866A4 EP3806866B1 Methods and materials for treating glycosylation disorders (2019)	UDP-galactose, UDP-glucose and derivatives, in the presence or absence of D-gal	The effect of D-gal has been studied in vivo on nine PGM1-CDG patients, but also in vitro on patient skin fibroblasts. Many chemical, biochemical, clinical parameters have been checked to assess the validity of the treatment. The efficacy of the administration of UDP-Gal in the presence of glucose or a derivative thereof (e.g., UDP-glucose) to efficiently correct the PGM1-CDG phenotype, has been assessed using PGM1-CDG patient skin fibroblasts. [64]	Disorder of multiple glycosylation pathways: • PGM1-CDG Phosphoglucomutase 1 Protein N-glycosylation: • PMM2-CDG Phosphomannomutase 2 • MPI-CDG Phosphomannose isomerase	Combined sugar supplementation therapy (UDP-glycans and D-galactose)
WO2022272056A2 Compositions and methods for treating PGM1 deficiency (2022)	AAV9 hPGM1 gene	AAV9-hPGM1 gene replacement prevents cardiac dysfunction in Pgm2 cKO mice. [68]	Disorder of multiple glycosylation pathways: • PGM1-CDG Phosphoglucomutase 1	Gene replacement therapy Recombinant adeno-associated virus (AAV) gene therapy
EP3175859A1 EP3175859B1 N-acetyl mannosamine for the treatment of a kidney-disease (2008)	N-acetyl mannosamine and derivatives	Gne^p.Met7l2Thr/p.Met7l2Thr^, Gne^p.Met7l2Thr/+^ and Gne^+/+^ mice were used to test the drugs. N-acetyl mannosamine and derivatives were useful for treating myopathies, muscular atrophy and/or muscular dystrophy and kidney conditions and diseases (e.g., those involving proteinuria and hematuria). [65]	Disorder of multiple glycosylation pathways: • GNE-CDG (UDP-N-Acetyl)-2-epimerase/N-Acetylmannosamine kinase	Substrate replacement therapy Administration of N-acetyl mannosamine and/or its derivatives promotes formation of sialic acid
CN104271125A WO2013109906A2 Method and formulation for treating sialic acid deficiencies (2013)	Sialic acid, oral administration in different formulations	27 patients were enrolled for a phase 1 study that was successful. Tests on animals (dogs) were conducted too. [66]	Disorder of multiple glycosylation pathways: • GNE-CDG (UDP-N-Acetyl)-2-epimerase/N-Acetylmannosamine kinase	Substrate replacement therapy Single or combined therapy was applied with other compounds (for example ManNAc) or derivatives of the sialic acid biosynthetic pathway. Sustained release formulation was also practiced Variations in the gene *GNE* cause a decrease in activity in either the isomerase or kinase domains, resulting in less formation of ManNAc-6-P and ultimately less Neu5AcA.
WO2019118486A1 Monosaccharide phosphoramidate prodrugs (2019)	Phosphoramidate prodrug of N-acetyl-D-mannosamine (ManNAc) 6-phosphate	Tests have been conducted to demonstrate that protecting groups were cleaved to restore the monophosphate sugar upon exposure to carboxypeptidase. Lec3 mutant CHO cells and GNE myopathy patient-derived myoblasts were treated with phosphoramidate prodrug of ManNAc 6-phosphate and the increase of free and total sialic acid recorded was higher than that obtained when ManNAc 6-phosphate has been was administered. [67]	Disorder of multiple glycosylation pathways: • GNE-CDG UDP-GlcNAc 2-epimerase/ManNAc kinase	Substrate replacement therapy Phosphoramidate derivative of sugars (N-acetyl-D-mannosamine 6-phosphate, mannose, etc.) enables the intracellular delivery of the monosaccharide
US2006276376A1 Increasing functional glycosylation of alpha-dystroglycan in the treatment of muscle degeneration (2006)	Glycosyltransferase (LARGE or LARGE2, or glycosyltransferases other than LARGE).	Direct injection of glycosyltransferase or exogenous construct harboring an expressible cDNA construct. FCMD myoblasts MEB fibroblasts WWS cells MCK-DG null mice expressing DG deletion mutant proteins.	Disorder of O-mannosylation: • LGMD2I limb-girdle muscular dystrophy linked to the FKRP gene MDC1D but also of FCMD, MEB, WWS, LGMD2I and other glycosyltransferase-deficient muscular dystrophies	LARGE expression can prevent muscle degeneration in various types of muscular dystrophy Increasing glycosyltransferase activity in the muscle of the subject would increase functional glycosylation of [alpha]-dystroglycan
JP2020510416A/ US2020317761A1 Multispecific binding molecules having specificity to dystroglycan and laminin-2 (2018)	A bispecific binding molecule comprising a first binding domain that binds an extracellular portion of α-dystroglycan and a second binding domain that binds laminin-2	In vivo studies on LARGEmyd-3 J/GrsJ mice. [71]	Disorder of O-mannosylation: α-dystroglycanopathy	Bispecific antibody which binds an extracellular portion of α-dystroglycan and laminin-2 Hypoglycosylation of alpha-dystroglycan results in a loss of binding of its ligands (such as laminin-2). This antibody promotes the binding between α-dystroglycan and laminin-2
US10456367B2 Compositions and methods for treating muscular dystrophy and other disorders (2018)	ribitol	p.Phe448Leu mice (containing the p.Phe448Leu variant in the fukutin-related protein gene) demonstrate a dystrophic phenotype similar to that of LGMD2I. Oral administration of ribitol increases levels of ribitol-5-phosphate and CDP-ribitol and restores therapeutic levels of F-α-DG in skeletal and cardiac muscles. [70]	Disorder of O-mannosylation: • FKRP-CDG α-dystroglycanopathy	Ribitol supplementation aims to circumvent ribitol shortage and ultimately the recovery of alpha-DG glycosylation
US10221168B1 Small-compound enhancers for functional O-mannosylation of alpha-dystroglycan, and uses thereof (2019)	Enhancer of O-mannosylation of alpha-dystroglycan	The enhancement of the functional O-mannosyl glycans of a-DG was tested on various cells: Chinese hamster ovary (CHO), mouse myoblasts (C2C12), normal human myoblasts (HSMB), human FKRP deficient myoblasts (FKRPD). [69]	Congenital muscular dystrophy characterized by reduced of O-glycosylation in the mucin-like domain of α-dystroglycan α -dystroglycanopathy	Enhancer of the functional O-mannosyl glycan (FOG) of α-DG on the cell surfaces
US2017368199A1 Methods and compositions for treating dystroglycanopathy disorders (2016)	Synthetic (optimized or not naturally occurring) polynucleotides encoding fukutin related protein (FKRP)	In vivo test in FKRP mutant mouse models. [73]	Disorder of O-mannosylation: • FKRP-CDG Fukutin-related protein	Gene replacement therapy Recombinant adeno-associated virus (AAV) gene therapy
CN110944656A WO2019008157A1 Novel polynucleotides encoding a human FKRP protein (2019)	Polynucleotides encoding human fukutin-related protein (FKRP) and containing variants that avoid complementary transcripts generated from the frameshift start codon	Gene transfer efficiency was evaluated in C57B16 mice. In vivo functional tests were conducted on HAS-FKRPdel mice. Optimization of human FKRP transgene improved FKRP expression and as a consequence ameliorated the efficacy of the treatment. [72]	Disorder of O-mannosylation: • FKRP-CDG Fukutin-related protein	Gene replacement therapy Recombinant adeno-associated virus (AAV9) gene therapy. FKRP-based gene replacement therapy
WO2021053124A1 Gene therapy expression system alleviating cardiac toxicity of FKRP (2021)	Expression system which ensures the production of a therapeutically effective amount of the protein in the target tissues (mainly the skeletal tissues) and a toxically acceptable amount of the protein in the heart.	The invention aims at alleviating or curing the devastating pathologies linked to a fukutin-related protein (FKRP) deficiency such as Limb-Girdle Muscular Dystrophy type 21 (LGMD2I).	Disorder of O-mannosylation: • LGMD2I Limb-girdle muscular dystrophy linked to the FKRP gene	Gene replacement therapy Recombinant adeno-associated virus (AAV) gene therapy. In order to alleviate toxicity, the construct may contain a target sequence of an miRNA expressed in the heart or a promoter sequence presenting a promoter activity at a toxically acceptable level or even no activity in the heart
WO2022147490A1 Optimized fukutin-related proteins (FKRT) and methods of use (2022)	Synthetic polynucleotide encoding a human FKRP	The evaluation of therapeutic effects of different vectors and different FKRP constructs was conducted on p.Glu310stop /p.Leu276Ile mice.	Disorder of O-mannosylation: • FKRP-CDG Fukutin-related protein	Gene replacement therapy Recombinant adeno-associated virus (AAV9) gene therapy
WO2022076556A2 Therapeutic adeno-associated virus delivery of fukutin related protein (FKRP) for treating dystroglycanopathy disorders including Limb Girdle 2i (LGMD2I) (2022)	AAV9 FKRP vector; the sequence has been codon optimized; viral vectors comprise nucleic acid encoding FKRP polypeptide operatively linked to a muscle-specific promoter	The AAV9 FKRP vector has been tested in the mouse model of LGMD2I. The strength of synthetic specific promoters was tested in H9C2 (a rat BDIX heart myoblast cell line). Determination of FKRP activity using LGMD2I patient- derived cell line.	Disorder of O-mannosylation: • LGMD2I Limb-girdle muscular dystrophy linked to the FKRP gene	Gene replacement therapy AAV gene therapy product candidate containing FKRP

**Table 3:** Drugs delivery tools for CDG

Patent id/Title/ earliest publication	Details of the patent
RS62641B1/EP3954360A2 Pharmaceutical preparation of carbohydrates for therapeutic use (2015)	‘The disclosure provides methods for preparation of carbohydrate replacement therapies (CRT) that include nanocarriers of carbohydrates and glycolipids for pharmaceutical delivery to cell interior, endoplasmic reticulum, and Golgi for treating CDG type I and CDG type II diseases as well as other metabolic disorders.’
CN113873998A/US2022184107A1 Liposomal formulations, and methods of using and preparing thereof (2022)	‘The disclosure provides phosphorylated carbohydrate replacement therapies (CRT) that include compositions of phosphorylated carbohydrates and phospholipids, as well as methods for preparing such compositions. Such compositions are suitable for pharmaceutical delivery of phosphorylated carbohydrates to cell interior, endoplasmic reticulum, and Golgi, and can be used for treating CDG type I and CDG type II diseases as well as other metabolic disorders.’

### Protein N-glycosylation (PMM2-CDG, MPI-CDG, ALG11-CDG)

Nutritional intervention with oral supplementation of sugars or their derivatives has been largely practised among CDG and is still today [31].

Oral mannose supplementation therapy was the first therapeutic approach for the PMM2-CDG, as it successfully restored glycosylation in patients’ fibroblasts [32]. However, no clinical improvement was recorded in PMM2-CDG children during the treatment, so this was dismissed for PMM2-CDG [33]. This treatment did not cause adverse effects on patients [34]; thus, in 1998, upon promising results obtained in vitro using MPI-CDG patient fibroblasts, mannose was orally administered to a newly identified MPI-CDG patient. His clinical symptoms disappeared, and his transferrin glycoprofile normalized (DE19758059A1, [35]). This therapeutic approach has proven to be an effective therapy for MPI-CDG patients [36, 37], and it has been approved both in the EU and the US. International consensus guidelines regarding managing MPI-CDG and oral administration of mannose have been recently proposed [38]. However, liver transplantation might be needed since limited results have been recorded as to the liver disease [39, 40].

PMM2-CDG is the most frequent CDG, with more than 1000 patients diagnosed worldwide. Seven out of 25 patents regarding CDG drugs or therapeutic approaches are related to this condition. It is a disorder of N-linked protein glycosylation, due to defective assembly and transfer of oligosaccharides to protein asparagine residues.

Biochemical characterization of wild-type and mutant PMM2 [41–45], and the knowledge of the molecular mechanisms underlying the disease, allowed the identification of (pro)drugs and the exploration of different therapeutic approaches, which have been an object of patents, too [14, 46, 47].

The first two patents pursued the substrate replacement therapy approach (SRT). The enzyme variants decrease GDP-mannose levels due to a reduced conversion of mannose-6P into mannose-1P. GDP-mannose plays an essential role in N-glycan biosynthesis. The approach explored by patents WO03104247A2 and US2009054353A1 featured the supplementation of mannose-1P. However, this monophosphate is highly polar and cannot diffuse through the cell membrane. On the other hand, hydrolytic enzymes (in the stomach, intestine, and plasma) cause the degradation of monosaccharide monophosphates, limiting their absorption and bioavailability. Thus, the attention moved to the production of derivatives of mannose-1P with increased lipophilicity, namely hydrophobically masked mannose-1P (the WO03104247A2, [48]) and mono-(mannopyranosyl-1), di(mannopyranosyl-1) and tri(mannopyranosyl-1) phosphates (the US2009054353A1, [49]). Endogenous nonspecific enzymes (such as esterases or hydrolases) would ensure the release of the free monophosphate sugar. Ultimately, this approach would permit bypassing the deficient PMM2 activity.

A second exciting approach is using MPI inhibitors to treat PMM2-CDG (WO2011116355A2). PMM2 and MPI compete for mannose-6P. The efficacy of the inhibition of MPI would push the flux of mannose-6P towards the production of mannose-1P [50]. The application of the MPI inhibitor successfully led to the diversion of mannose-6P towards PMM2 and improved the defective N-glycosylation, at least in pre-clinical studies.

A galactose supplementation therapy has also been explored for PMM2-CDG, and an open-label pilot trial has been conducted, but the suitability of this approach has not been fully addressed (EP3806866A1, NCT02955264, [51]).

Despite promising results in vitro, substrate replacement therapy and MPI inhibition have not been approved for PMM2-CDG. However, carbohydrate replacement therapy could become feasible thanks to the refinement of the drug delivery systems that were the object of two patents listed in Table 3. Specifically, both the selected patents regarding the tools for drug delivery (EP3954360A2 and US2022184107A1) describe the preparation of liposomes designed to deliver mannose-1-phosphate. A phase 2 clinical trial of GLM101 for treating PMM2-CDG is underway (NCT05549219).

Recently, another therapeutic approach has been attempted in diseases caused by missense variants where a mutation causes a destabilization, i.e., the use of pharmacological chaperones (PCs) [52]. In these cases, an accurate evaluation of the effect of missense variants on the protein functioning or stability is needed, as it would be difficult to distinguish between disease and non-disease variants [53–55] clearly.

In CDG research, this PC therapy is still in its early steps of in vitro investigation. For PMM2-CDG, extensive screening of commercial molecules and rational drug design led to the identification of putative PCs (EP3275863A1, [56, 57]).

The most recent patents regarding drugs to treat PMM2-CDG patients relate to the application of aldose reductase inhibitors (patents WO2020040831A1 [58], US2022017535A1, and WO2021071965A1).

A high-throughput screening of commercially available drugs led to identifying and rationalising this class of compounds for therapeutic purposes. The use of patient-derived fibroblasts, as well as worm and yeast models, ensured the success of this study. Among the AOR inhibitors, epalrestat gained much attention. It is commonly used for treating diabetic neuropathy in Japan and is the only antidiabetic aldose reductase inhibitor approved for use in humans. A phase 3 clinical trial is currently in progress (NCT04925960). The rationale for its efficacy is that it may shunt glucose from the polyol pathway to glucose-1,6-bisphosphate, which is an endogenous stabilizer and coactivator of PMM2 homo- or hetero-dimerization [27, 57, 59, 60].

Dietary supplementation and organ transplantation currently represent the only curative therapies available for CDG; most patients can only receive symptomatic and preventive treatments. In this frame, the therapeutic application of cannabidiol (CBD) could represent another available tool. In 2018, the FDA approved CBD for treating seizures associated with rare epilepsy syndromes [61, 62]. As an expansion of this application, CBD significantly reduced the number of seizures in an ALG11-CDG patient (GB2597315A).

### Disorders of multiple glycosylation pathways (PGM1-CDG and GNE-CDG)

Nucleotide sugars are the building blocks of glycans; several therapeutic approaches rely on treatments that aim to increase the intracellular concentration of these molecules. This approach produced some patents, mainly regarding disorders of multiple glycosylation pathways such as PGM1-CDG, PGM3-CDG and GNE-CDG.

An example of this approach is the combined therapy of uridine prodrug and sugars (WO2016028894A1), an approach tested in three patients with a different CDG (GNE-myopathy, PGM1-CDG, and DPAGT1-CDG); the coadministration of uridine triacetate and the specific sugar caused increased intracellular concentrations of the UDP-sugar. In a second approach, a sugar supplementation therapy combined UDP-glycans and D-galactose (PMM2-CDG and MPI-CDG) (EP3806866A1). This approach looks like an evolution of the dietary intervention through monosaccharide supplementation, primarily explored in CDG, although not all trials have succeeded [63]. Galactose therapy trials significantly improved biochemical abnormalities, but no clinical progression data have been reported. A metabolomic study shed light on the mechanism of PGM1-CDG and suggested that: “The direct administration of nucleotide sugars may be a more effective and less onerous form of treatment for affected individuals than galactose therapy.“ Such an approach could represent a starting point for other CDG related to nucleotide sugar metabolism and transport [64].

Substrate replacement therapies have also been patented for GNE-CDG. Variations in *GNE* cause a decrease in activity in either the isomerase or kinase protein domains, resulting in less formation of ManNAc-6-P and, ultimately, less Neu5AcA (sialic acid). In one study, N-acetyl mannosamine and derivatives proved helpful in treating myopathies, muscular atrophy or muscular dystrophy and kidney conditions and diseases in mice (EP3175859A1, [65]). On the other hand, in a phase 1 study, sialic acid was administered to patients (WO2013109906A2, [66]). In a third approach, a prodrug - a phosphoramidate derivative of ManNAc 6-phosphate - has been preferred to the monosaccharide monophosphate (WO2019118486A1, [67]).

Gene therapy is also starting to be applied in CDG, as described for PGM1-CDG (WO2022272056A2, [68]).

### O-glycosylation (α-dystroglycanopathies)

Dystroglycanopathies are a subset of muscular dystrophies due to reduced O-glycosylation in α-dystroglycan with diminished laminin-binding activity. Specific molecules can enhance this binding, as it happens for a bispecific antibody. It comprises a first binding domain that binds an extracellular portion of α-dystroglycan and a second binding domain that binds laminin-2 [69]. This approach is the object of patent US10221168B1. The unexpected discovery that ribitol can restore or enhance functional glycosylation of mainly α-dystroglycan led to the application of a sugar supplementation therapy that had also been a subject of a patent (US10456367B2, [70]). Also, small molecules enhancing functional O-mannosylation of α-dystroglycan have been identified (US10221168B1, [71]). These compounds proved active in several applications; they could improve the functional O-mannosylation of α-dystroglycan on B421 cells (partially deficient in DPM2 with a point variant) and FKRP defective cells. DPM2-CDG is a disorder of multiple glycosylation pathways, while FKRP-CDG is a disorder of O-mannosylation.

Gene replacement therapy for FKRP-CDG received great attention. Four patents have been produced (US2017368199A1, [72]; WO2019008157A1, [73]; WO2022147490A1; WO2022076556A2). They all describe the application of adeno-associated virus (AAV9) gene therapy using optimized polynucleotides encoding the fukutin-related protein. Specific constructs have also been studied to produce therapeutical and toxically acceptable levels of protein in the heart (WO2021053124A1).

Two clinical trials are in progress regarding gene therapy for FKRP-CDG (NCT05224505 and NCT05230459).

## Diagnostic tools for CDG

Table 4 lists patents concerning diagnostic tools for CDG.

**Table 4:** Diagnostic tools for CDG

Patent id/Title/earliest publication	Key features	Application
US5432059A Assay for glycosylation deficiency disorders (1995)	The method of the invention relies on the enzymatic derivatization (by using sialyltransferases and galactosyltransferases) of carbohydrate-deficient glycoproteins in samples of body fluids obtained from subjects with metabolic disorders. In this way fluoresceinylated monosaccharides can be incorporated into the carbohydrate-deficient glycoprotein target. Fluorescence emission from the reglycosylated glycoprotein can be measured.	Transferrin and other glycoproteins that have altered patterns of glycosylation in alcohol abusers or in subjects with genetic syndromes characterized by carbohydrate-deficient serum glycoproteins
US5993626A Capillary electrophoresis of transferrin glycoforms (1999)	Capillary electrophoresis is used to resolve and detect transferrin glycoforms that are indicative of pathologic states.	Chronic alcoholism and carbohydrate-deficient glycoprotein syndrome
US2002055184A1 Systems for detecting analytes (2001)	“The invention is based on the discovery that systems that include an affinity cartridge and a mass spectrometer are useful for detecting analytes such as transferrin, from a biological sample. When measuring CDTs, neuraminidase treated transferrin can be used as an internal standard, and can be applied to a membrane of affinity membrane cartridge, which is coated with antibodies having specific binding affinities for transferrin.”	Chronic alcoholism and carbohydrate-deficient glycoprotein syndrome, other inborn errors of metabolism
EP3117217A1 WO2015135900A1 Analytical method for the identification of at least one glycoform of the transferrin protein (2015)	“The invention refers to an analytical method for the identification of at least one transferrin glycoform and/ or isoform and/ or sialoform, and in particular of those known as carbohydrate-deficient transferrin, possibly present in a complex biological matrix, by protein functionalization with a source of lanthanide 3 + ion.”	Carbohydrate-deficient transferrin glycoforms used as a diagnostic marker
WO0033076A1 Diagnosis of human glycosylation disorders (2000)	The samples containing glycoconjugates are contacted with a diagnostic reagent that consists of a binding component (for example lectins, antibodies, etc.) and a label (such as fluorescent dyes, radiolabels, etc.). The ability of the diagnostic reagent to bind to the glycoconjugates in the sample is indicative of the presence or absence of the glycosylation disorder.	Carbohydrate Deficient Glycoprotein Syndromes
WO0192890A1 Methods for the analysis of picomole amounts of carbohydrates (2001)	“The invention essentially overcomes the problems encountered in downscaling (miniaturizing) the analysis of underivatized carbohydrates and glycoconjugates to the subpicomole level.”	Carbohydrate Deficient Glycoprotein Syndromes
US2006216766A1 Assay for protein isoforms (2004)	The method relies on the use of a proteolytic enzyme that hydrolyses the protein of interest producing a specific peptide pattern that is characteristic of the glycosylation profile of the target protein. The fragments may be detected by methods which require the use of specific binding partners, or by chromatography, mass spectrometry, NMR, etc.	Transferrin isoforms in patients with carbohydrate-deficient glycoprotein syndromes (CDGS) or congenital disorders of glycosylation (CDG) Other clinically relevant proteins exist in differently glycosylated isoforms, including glycosylated markers for cancers and other diseases
KR20100098500A US8877454B2 Apparatus for auto-pretreating sugar chain (2009)	“The invention provides an automatic oligosaccharide chain pre-treatment apparatus which is capable of performing purification at high speed and with high accuracy by automating processing steps.”	The profile of the whole serum N-glycan can distinguish CDG from healthy controls, and also certain subgroups of CDG from other subgroups of CDG
US2014271615A1 Hyposialylation disorders (2014)	The method includes measuring the ratio between the amount of monosialylated Thomsen-Friedenreich antigen and the amount of non-sialylated Thomsen-Friedenreich antigen in a biological sample (plasma or serum sample). The same measurement can be used for determining the effectiveness of a therapeutic agent.	Hyposialylation disorders (GNE myopathy and other CDG)
US2022291236A1 Detection of dystroglycan (2022)	Analytical methods for simultaneous detection of αDG and glycosylated αDG for dystroglycanopathy patient biopsies.	Hypoglycosylation of α-DG Limb-Girdle Muscular Dystrophy (LGMD2i) is a dystroglycanopathy caused by partial loss of function variants in the FKRP gene
WO9849324A2 WO9849324A3 Carbohydrate-deficient glycoprotein syndrome type I (1998)	Identification of the genetic defect associated with carbohydrate-deficient glycoprotein syndrome type I, and the possibility to use this finding for diagnosis and other purposes.	PMM2-CDG
WO0236757A2 Enzymes involved in glycoprotein and glycolipid metabolism (2002)	“The invention provides human enzymes involved in glycoprotein and glycolipid metabolism (GLYCOS) and polynucleotides which identify and encode GLYCOS. The invention also provides expression vectors, host cells, antibodies, agonists, and antagonists. The invention also provides methods for diagnosing, treating, or preventing disorder associated with aberrant expression of GLYCOS.”	SLC39A8-CDG SLC35C1-CDG SLC35A2-CDG PIGV-CDG PIGT-CDG PIGS-CDG PIGO-CDG PIGG-CDG
WO2004015110A1 Sugar chain synthase gene (2004)	Identification of genes involved in N-linked oligosaccharide synthesis in human ER. The function of the genes was obtained through complementation in yeast strains bearing a variants or deletions in the homologous genes.	ALG8-CDG ALG9-CDG ALG10-CDG ALG11-CDG ALG12-CDG
WO2006094344A1 Methods and agents for regulating cellular interactions and development (2006)	“Glycosylation pathways comprising PGM3 substrates play a key role in regulating hematopoietic stem cells.” “Mutations in Pgm3 cause a global reduction in UDP-GlcNAc levels and that despite the general importance of glycosylation in all cell types, incremental changes in UDP-GlcNAc levels selectively affect the modification of specific proteins, leading to a graded series of pathological changes.” PGM3 has been identified “as an important mediator for the in vitro or in vivo regulation of cellular interactions and development, in particular of stem cells and their subsequent lineages.” [76] Later, PGM3-CDG has been described as a severe infancy-onset immunodeficiency. [77, 90, 91]	PGM3-CDG
EP2905621A1 Means and methods for diagnosing and treating cdg caused by a deficiency of PGM1 (2015)	PGM1 deficiency is a mixed CDG. An appropriate enzymatic test measures the activity on cell extracts. Effects associated with this defect have been described both in patients and in cell lines. Among others, the efficacy of galactose supplementation has been described. [79]	PGM1-CDG
WO2022103815A1 Polyol biomarkers for congenital disorders of glycosylation (2022)	Elevated polyols as markers in PMM2-CDG. Patients with MAN1B1-CDG, ALG6-CDG, SLC39A8-CDG, MOGS- CDG, CDG type IIx, NANS-CDG, PIGS-CDG, and ALG8-CDG also demonstrated elevated levels of polyols. These biomarkers can be used to evaluate the efficacy of a treatment. [92]	PMM2-CDG MAN1B1-CDG ALG6-CDG SLC39A8-CDG MOGS- CDG CDG type IIx NANS-CDG PIGS-CDG ALG8-CDG
CN110997906A WO2018226560A1 B4GALT1 variants and uses thereof (2018)	B4GALT1 may have a serine instead of an asparagine at the position 352. This p.Asn352Ser variant is protective against one or more cardiovascular conditions. B4GALT1 may be used to diagnose a patient’s risk of developing cardiovascular conditions.	Cardiovascular disorders

Serum transferrin isoelectrofocusing (Tf IEF) is still the method of choice for screening N-glycosylation disorders associated with sialic acid deficiency. Initially, the test was introduced for the screening of chronic alcoholism. Serum transferrin is only N-glycosylated, and the bulk of it carries four sialic acids and, thus, four negative charges. Capillary zone electrophoresis is a valuable alternative screening method, but abnormal results have to be controlled by Tf IE [74] (US5993626A).

As expected, many other serum glycoproteins besides transferrin show altered isoforms in CDG. Overall a wide range of methodologies has been explored and developed, such as the enzymatic derivatization of carbohydrate-deficient glycoproteins with fluoresceinylated monosaccharides and measurement of the fluorescence of the re-glycosylated glycoproteins (US5432059A), study of the interaction of glycoproteins with lectins or antibodies (WO0033076A1), and the MS or NMR of the specific peptide pattern obtained by enzymatic hydrolysis of the glycoprotein of interest (US2006216766A1). In addition, methods ensuring a higher sensitivity or a lesser sample handling have also been patented (such as WO0192890A1 and US8877454B2).

Another way to assess alterations of glycosylation profiles is the evaluation of the *ratio* of the amount of mono-sialylated to non-sialylated Thomsen-Friedenreich antigen in a biological sample, as described in US2014271615A1. Also, a specific method for simultaneous detection of α-DG and glycosylated α-DG for dystroglycanopathy patient biopsies has been patented (US2022291236A1).

Serum Tf IEF has some limitations; moreover, it is not a suitable biomarker for in vitro studies on cell models such as fibroblasts. Recently, polyols were identified as robust biomarkers of PMM2-CDG and several other CDG (WO2022103815A1).

However, genetic analysis is the most reliable diagnostic [75].

The discovery of the defect associated with carbohydrate-deficient glycoprotein syndrome type I or Jaeken disease, subsequently renamed PMM2-CDG, was patented in 1998 (WO9849324A2). Several glycoprotein and glycolipid metabolism enzymes were patented in 2002 (WO0236757A2).

PGM3 has long been considered a biomarker for forensic purposes. In 2006 it was described “as an important mediator for the in vitro or in vivo regulation of cellular interactions and development, in particular of stem cells and their subsequent lineages” (WO2006094344A1, [76]). PGM3 deficiency was first described in 2014 in patients with hyper-IgE syndrome phenotype characterized by recurrent infections, atopy, and elevated serum IgE [77, 78]. In 2015, PGM1-CDG was described at the clinical, biochemical and molecular levels, together with a possible therapeutic strategy based on the dietary supplementation of galactose (EP2905621A1, [79]).

Complementation experiments in yeast addressed the function/dysfunction of several genes involved in N-glycosylation, such as *ALG8*, *ALG9*, *ALG10*, *ALG11*, and *ALG12* (WO2004015110A1).

## Discussion

Among the most recent patents regarding drugs and therapeutic approaches, some led to clinical trials that are currently in progress, for example, epalrestat (NCT04925960), GLM101 (NCT05549219), and Adeno-Associated Virus vector carrying the human FKRP transgene (GNT0006, NCT05224505). Other patented drugs are still under development, such as the amide and urea derivatives that could act as pharmacological chaperones (EP3275863A1).

Other drugs under clinical trials were not traceable back to any of the selected patents. This happened, for example, for acetazolamide (for PMM2-CDG, NCT04679389), oral GlcNAc Supplementation (for NGLY1 deficiency, NCT05402345), ManNAc (for GNE myopathy, NCT04231266), AVTX-803 (for Leukocyte Adhesion Deficiency type II, NCT05462587O). Moreover, other drugs, prodrugs or lead compounds are under pre-clinical studies: celastrol (a proteostasis regulator, tested for PMM2-CDG), palovarotene (Sohonos™, an orally bioavailable selective retinoic acid receptor (RAR)γ agonist, tested for EXT1/EXT2-CDG), glucose 1,6 bisphosphate (an activator of the PMM2 that also acts as a pharmacological chaperone, tested for PMM2-CDG), clodronate (inhibitor of PMM1 that could have an indirect beneficial effect on PMM2 activity, tested for PMM2-CDG).

The commercialization of patented molecules is an exciting issue. To take a deeper insight, we focused on patents submitted by companies and screened the web to find information about the patent’s follow-up. The number of newly commercialized drugs is meagre. Substrate replacement therapy with mannose-1-phosphate has been produced as GLM101 by Glycomine; it has received Orphan Drug Designation (ODD) in the US and Europe and Rare Pediatric Disease Designation (RPDD) in the US and is in clinical trial phase 2 (NCT05549219) [80]. AT007, an aldose reductase inhibitor, is currently commercialized as the orphan drug Govorestat by Applied Therapeutics; Govorestat has not yet advanced in clinical trial for PMM2-CDG but is currently in clinical trial for different diseases (galactosemia, NCT04902781, and SORD Deficiency, NCT05397665) [81].

Repurposed drugs provide a different topic, being approved and then repositioned for CDG. For example, this is the case for Epalrestat, the first aldose reductase inhibitor patented by Perlara for use in PMM2-CDG (WO2020040831A1) and currently undergoing clinical trial phase 3.

Interestingly, many of these drugs derive from repositioning, a precious strategy in rare diseases [82–84]. With this approach, knowing the patterns involved in the drug’s action helps find different applications, either on known targets or off-targets [85–87]. Serendipity is typical in drug repurposing, but on the other hand, data provided by ‘omics’ experiments contain information about the differential regulation of genes by specific treatments. For example, while investigating the effect of acetylsalicylic acid on Fabry patient-derived fibroblasts, Monticelli and co-workers recorded a strong down-regulation of the COG5 protein (0.103 ratio ASA/control [88]).

Blood tests for diagnosis are available, but molecular genetic testing represents the final diagnostic tool. Anyway, screening methods are not 100% reliable and even enzyme measurements could be debatable in mild cases, like in PMM2-CDG, but in many cases genetic results are not conclusive for the diagnosis and a biochemical or functional confirmation is essential for establishing the diagnosis. Therefore, the development of biomarkers, diagnostic and screening tools is of utmost importance. Many people with CDG are undoubtedly misdiagnosed [7], particularly those with mild or atypical phenotypes [89].

The CDG illustrate that incorrect glycosylation is associated with a bewildering broad phenotypic spectrum. However, the absence of a genotype-phenotype correlation shows the remarkable role of genomic variants in the development of the disease [26–29]. In this regard, it is interesting to note that one of the patents concerns *B4GALT1* and describes the protective effect of the p.Asn352Ser variant against one or more cardiovascular conditions (Table 3).

Our data clearly show the increasing interest in CDG by the Scientific Community. In the last few decades, many advances have been pursued. Nevertheless, besides the newly acquired knowledge, few drugs moved to clinical trials, and none of these has been approved, apart from the orphan drug designation. Among the reasons behind these data, the lack of funding for rare diseases is undoubtedly a significant issue [7]. In our opinion, cooperation among the different stakeholder groups (i.e. researchers, clinicians, families and companies) would be a unique possibility to boost CDG research and reach the goal of approved therapies as soon as possible.

## Conclusion

Our research on CDG patents identified 25 documents regarding drugs or therapeutic approaches, 17 regarding diagnostic tools and two regarding drug delivery tools. These patents (regarding numbers and specificities) can be considered indicators of the attention paid to the CDG and the success gained through clinical and research activities.

### Electronic supplementary material

Below is the link to the electronic supplementary material.

Supplementary Material 1

Supplementary Material 2

Supplementary Material 3

Supplementary Material 4

Supplementary Material 5

## Data Availability

not applicable.

## Abbreviations

α-DG: α-dystroglycan
ALG6: Dolichyl pyrophosphate Man9GlcNAc2 alpha-1,3-glucosyltransferase
ALG8: Probable dolichyl pyrophosphate Glc1Man9GlcNAc2 alpha-1,3-glucosyltransferase
ALG9: α-1,2-mannosyltransferase ALG9
ALG10: Dol-P-Glc:Glc(2)Man(9)GlcNAc(2)-PP-Dol alpha-1,2-glucosyltransferase
ALG11: GDP-Man:Man(3)GlcNAc(2)-PP-Dol alpha-1,2-mannosyltransferase
ALG12: Dol-P-Man:Man(7)GlcNAc(2)-PP-Dol alpha-1,6-mannosyltransferase
B4GALT1: β-1,4-galactosyltransferase 1
CDG: Congenital Disorders of Glycosylation
COG5: Conserved oligomeric Golgi complex subunit 5
DPAGT1: UDP-N-acetylglucosamine–dolichyl-phosphate N-acetylglucosaminephosphotransferase
EXT1: Exostosin-1
EXT2: Exostosin-2
FCMD: Fukuyama congenital muscular dystrophy
FKRP: Ribitol 5-phosphate transferase
GNE: Bifunctional UDP-N-acetylglucosamine 2-epimerase/N-acetylmannosamine kinase
LMGD2I: Autosomal recessive form of limb girdle muscular dystrophy (LGMD)
MAN1B1: Endoplasmic reticulum mannosyl-oligosaccharide 1,2-alpha-mannosidase
MCK-DG: Muscle creatine kinase/α-dystroglycan
MDC1D: Congenital muscular dystrophy type 1D
MEB: Muscle eye brain disease
MOGS: Mannosyl-oligosaccharide glucosidase
MPI: Mannose phosphoisomerase
NANS: Sialic acid synthase
NGLY: Peptide-N(4)-(N-acetyl-beta-glucosaminyl)asparagine amidase
PGM1: Phosphoglucomutase-1
PGM3: Phosphoglucomutase-3
PIGG: GPI ethanolamine phosphate transferase 2
PIGO: GPI ethanolamine phosphate transferase 3
PIGS: GPI transamidase component PIG-S
PIGT: GPI transamidase component PIG-T
PIGV: GPI mannosyltransferase 2
PMM2: Phosphomannomutase-2
SLC35A2: UDP-galactose translocator
SLC35C1: GDP-fucose transporter 1
SLC39A8: Metal cation symporter ZIP8
Tf IEF: Serum transferrin isoelectrofocusing
WWS: Walker-Warburg syndrome
